# Utility of brain imaging in pediatric patients with a suspected accidental spinal injury but no brain injury-related symptoms

**DOI:** 10.1007/s00381-024-06298-8

**Published:** 2024-01-27

**Authors:** Aapo Sirén, Mikko Nyman, Johanna Syvänen, Kimmo Mattila, Jussi Hirvonen

**Affiliations:** 1 Department of Radiology, University of Turku and Turku University Hospital, Kiinamyllynkatu 4-8, 20520 Turku, Finland; 2 Department of Pediatric Orthopedic Surgery, University of Turku and Turku University Hospital, Turku, Finland; 3 Medical Imaging Center, Department of Radiology, Tampere University and Tampere University Hospital, Tampere, Finland

**Keywords:** Brain, Spine, Trauma, Magnetic resonance imaging, Pediatrics, Injury

## Abstract

**Purpose:**

Imaging is the gold standard in diagnosing traumatic brain injury, but unnecessary scans should be avoided, especially in children and adolescents. Clinical decision-making rules often help to distinguish the patients who need imaging, but if spinal trauma is suspected, concomitant brain imaging is often conducted. Whether the co-occurrence of brain and spine injuries is high enough to justify head imaging in patients without symptoms suggesting brain injury is unknown.

**Objective:**

This study aims to assess the diagnostic yield of brain MRI in pediatric patients with suspected or confirmed accidental spinal trauma but no potential brain injury symptoms.

**Methods:**

We retrospectively reviewed the medical and imaging data of pediatric patients (under 18 years old) who have undergone concomitant MRI of the brain and spine because of acute spinal trauma in our emergency radiology department over a period of 8 years. We compared the brain MRI findings in patients with and without symptoms suggesting brain injury and contrasted spine and brain MRI findings.

**Results:**

Of 179 patients (mean age 11.7 years, range 0–17), 137 had symptoms or clinical findings suggesting brain injury, and 42 did not. None of the patients without potential brain injury symptoms had traumatic findings in brain MRI. This finding also applied to patients with high-energy trauma (*n* = 47) and was unrelated to spinal MRI findings.

**Conclusion:**

Pediatric accidental trauma patients with suspected or confirmed spine trauma but no symptoms or clinical findings suggesting brain injury seem not to benefit from brain imaging.

## Introduction

Pediatric traumatic brain injury is a significant worldwide health problem [1]. Emergency department (ED) visits because of suspected brain injury are common [2], and both ED visits [3] and confirmed mild traumatic brain injuries [4] have an emerging trend in the pediatric population, as well as overall rates of trauma-related ED visits, including visits because of cervical spine traumas [5].

With every child in the ED suspected of having accidental head trauma, the essential question is to scan or not to scan? [6] With many patients, clinical decision-making tools such as PECARN [7], CATCH [8], or CHALICE [9] help answer the question with high sensitivity [10]. In clinical practice, children primarily suspected of having a spine injury but no risk factors regarding brain injury often undergo concurrent brain and spine imaging. Still, it is unknown whether spine trauma is an individual risk factor for brain injury in accidental trauma. In non-accidental trauma, co-occurrence of brain and spine injuries is shown to be relatively prevalent [11–14], but these children are mostly very young, and the injury mechanisms differ from those in accidental injuries.

We have been able to use MRI widely in pediatric emergency spine trauma imaging [15]. If a child is undergoing a spine MRI because of trauma, a head MRI has often been performed concurrently and vice versa, but as far as we know, there is no scientific evidence justifying this practice in asymptomatic patients. Therefore, this study aimed to assess the utility of the concurrent use of brain and spine MRI in pediatric trauma patients without symptoms suggesting brain injury. We hypothesized that if the patient had no brain injury-related symptoms, the additional yield of a concurrent brain MRI with spine MRI was low, even in the presence of spine injury.

## Materials and methods

The charts of the under-18-year-old patients who had undergone an emergency spinal MRI at our Emergency Radiology Department between April 1, 2013, and August 31, 2021, were reviewed retrospectively. Our hospital is a tertiary care referral center for approximately 470,000 people. The inclusion criteria for the study sample were (1) emergency spinal MRI and (2) concurrent brain MRI. The patients with (1) primary MRI indications other than blunt trauma and (2) patients with trauma but no Pediatric Emergency Care Applied Research Network (PECARN) risk factors [16] or reasoned clinical suspicion for thoracolumbar spine injury (based on symptoms and clinical findings) were excluded. At our institution, the diagnostic workup of children with a suspicion of non-accidental trauma is carried out in the Department of Pediatric Radiology, and these patients are, therefore, not included in this study.

The radiology information system (RIS) was reviewed to extract imaging reports with MRI findings. Medical records were reviewed for injury mechanisms and demographic and clinical variables. The symptoms and findings signaling a possible head injury—including headache, confusion, momentarily or persistent altered mental state including unconsciousness, dizziness, amnesia, seizure, nausea, vomiting, gait disturbances, sensory function alterations, hemiparesis, irritability, and cranial nerve findings, e.g., anisocoria and diplopia—were carefully noted. The usual clinical decision rules (PECARN, CATCH, or CHALICE) for the brain injury risk assessment were not used, even retrospectively, because the relevant information was primarily not collected in a structured manner. The Glasgow Coma Scale (GCS) [17] nor the Pediatric Glasgow Coma Scale (pGCS) [18] was neither systematically found from the records, although the level of consciousness was always assessed. In the subgroup with high-energy trauma, the injury mechanism had been primarily interpreted to be severe enough to trigger the initial evaluation and care with standardized trauma protocol by the trauma team. The trauma mechanisms in this subgroup included the following: (1) car accident with a speed of at least 60 km per hour, (2) pedestrian struck by car, (3) bicyclist struck by car, bicycle accident with known high speed or with unknown circumstances and worrisome clinical findings (e.g., altered consciousness, unstable hemodynamics, dislocated fractures), (4) motorcycle accident, and (5) fall from a height of two meters or more.

An on-call physician, usually a pediatric orthopedic surgeon, trauma surgeon, or neurosurgeon, referred the MRI scans based on clinical judgment. All patients included in the study sample had at least one PECARN risk factor for cervical spine trauma [16] or equivalent symptoms or findings regarding the thoracolumbar spine. In our department, MRI has been widely used as a first-line imaging modality in suspected spinal trauma (15). For 142/179 (79.3%) patients in the current study sample, MRI was the first spinal imaging performed because of spinal trauma. Of the patients with spinal CT before MRI, 22/37 (59.5%) had CT findings leading to the MRI referral. With the patients having undergone unremarkable CT (15/37, 40.5%), MRI was performed because of an altered level of consciousness (7/15), severe spinal pain (7/15), or neurologic deficit (1/15). However, none of the patients without traumatic findings in spinal CT had spinal MRI findings altering the treatment. No conventional spinal radiographs were obtained at the emergency department.

MR imaging was performed in the emergency radiology department using a Philips Ingenia 3-T system with a Philips dStream coil system (Philips Healthcare, Best, Netherlands). The brain MRI protocol included at least the following sequences: axial T2-weighted, isotropic 3D T1-weighted, isotropic 3D FLAIR, axial diffusion-weighted (DWI), and axial susceptibility-weighted (DWI) sequences. The spinal MRI protocol included sagittal T1-weighted, sagittal and axial T2-weighted, and sagittal and coronal short tau inversion recovery (STIR) sequences. In selected cases, the dedicated small field of view (FOV) proton density- and T2-weighted series were used for the craniocervical junction (occipital bone–second cervical vertebra, C0–C2).

The seniority of the radiologist reporting the MRI studies was as follows: Fellowship-trained subspecialists in neuro- or emergency radiology (with > 7 years of experience in radiology) reported 144/179 (80.4%) of the MRIs, other consultant radiologists (with > 5 years of experience in radiology) reported 34/179 (19.0%), and one MRI (0.6%) was reported by a radiologist in training (with > 3 years of experience).

Of the whole study sample, 161/179 patients (89.9%) were scanned fully awake. Of the patients who were sedated or anesthetized during the MRI, 6/18 (33.3%) were already intubated because of decreased consciousness or non-neurological injuries requiring anesthesia and intubation. Light sedation with spontaneous breathing was used with 11/173 (6.4%) previously awake patients to perform an MRI, and only one out of 173 patients (0.6%) was intubated before the MRI. However, this patient had severe TBI and was kept intubated at the ICU also after the scan. The age range of patients sedated or intubated to perform MRI was 0–10 years, with the median being 4 years. The standard practice was to perform an MRI with the patient being awake whenever viable, without definite rules on which age groups to be sedated. The need for anesthesia was assessed by the referring physician case by case. If the examination could not be performed awake, the radiographers requested reassessment.

The results are expressed as the number of cases (*n*), percentage, mean, median, range, and standard deviation (SD). Proportions of categorical variables were compared with the Pearson chi-square (*X*^2^) test and Fisher’s exact test. *P*-values < 0.05 were considered statistically significant. The statistical analyses were performed using the IBM SPSS Statistics Package for Mac (version 29, IBM Corporation, Armonk, NY).

We obtained permission from the hospital district board, but institutional ethical review board approval and written patient consent were not needed for this retrospective study.

## Results

**Fig. 1:**
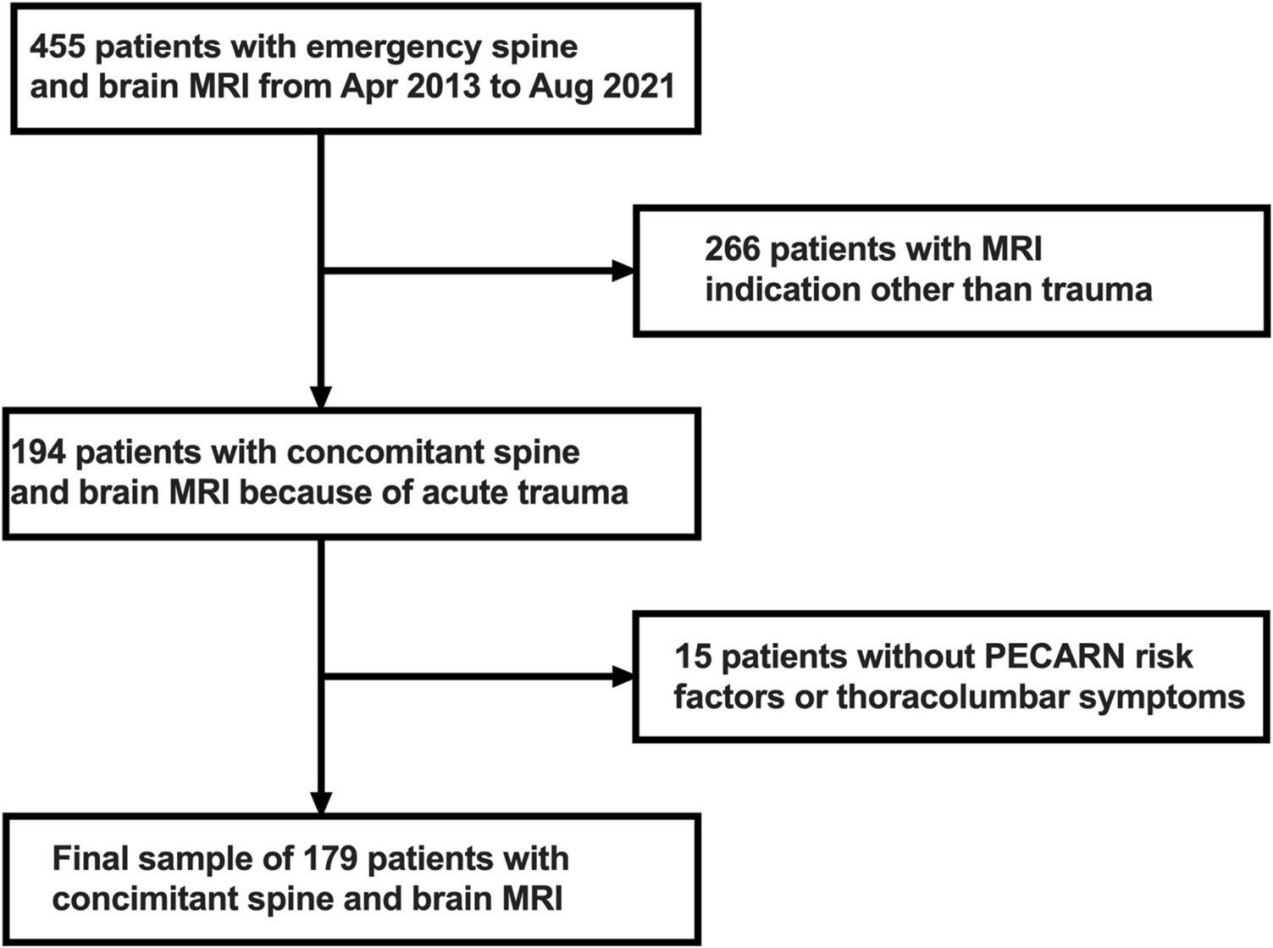
Flowchart of patient inclusion and exclusion

We found 455 patients meeting the inclusion criteria. After excluding 266 patients with MRI indications other than trauma and 15 patients with no PECARN risk factors for cervical spinal trauma or symptoms suggesting thoracolumbar injury, the total study sample included 179 patients (Fig. 1).

The mean age of the patients was 11.7 years, the median was 13 years, and the range was 0–17 years. The sex ratio was almost even. The demographic characteristics, injury mechanisms, and imaging findings are demonstrated in Table 1. Most patients (136/179, 76%) had symptoms or findings suggesting brain injury, while 43/179 (24%) did not (Table 1). The most common injury mechanism was falling. The cervical spine was the primary suspected injury level in 96% (171/179) patients, and 4% (8/179) were primarily suspected of having thoracolumbar spine injury.

**Table 1:** Demographic characteristics, injury mechanisms, the occurrence of probable brain injury symptoms, and MRI findings in the study population

Number of patients	179
Age, mean (SD), median (range)	11.7 (4.4), 13 (0–17)
Female *n* (%)	93 (52)
*Injury mechanism*	*n* (%)
Fall	71 (39.7)
Motor vehicle accident^a^	33 (18.4)
Sports	33 (18.4)
Bicycle or kick scooter	12 (6.7)
Horseback riding	11 (6.1)
Violence^b^	8 (4.5)
Trampoline	6 (3.4)
Miscellaneous^c^	5 (2.8)
Potential brain injury symptoms, *n* (%)	137 (76.5)
No potential brain injury symptoms,* n* (%)	42 (23.5)
*Incidence of traumatic findings on brain MRI*	*n* (%)
Diffuse axonal injury	11 (6.1)
Hemorrhagic contusion	11 (6.1)
Skull or skull base fracture	8 (4.5)
Epidural hematoma	6 (3.4)
Subdural hematoma	4 (2.2)
Subarachnoidal hemorrhage	1 (0.6)
Intraventricular hemorrhage	1 (0.6)
*Incidence of traumatic findings on spine MRI*	*n* (%)
Ligamentous injury	20 (11.2)
Osseous injury	19 (10.6)
Soft tissue edema only	11 (6.1)
Spinal cord injury	1 (0.6)
Additional head CT, *n* (%)	20 (22.3)
Additional spine CT, *n* (%)	42 (23.5)

^a^Including pedestrians struck by a car

^b^Patients with suspected abusive trauma are not included

^c^Diving, hanging, unknown trauma

Of the sample population, 14% (25/179) had traumatic findings in brain MRI. The findings included epidural hematomas, subdural hematomas, traumatic subarachnoidal hemorrhages, intraventricular hemorrhages, hemorrhagic contusions, and diffuse axonal injuries (Table 1). Skull fractures were found in nine patients (5%).

All patients with traumatic brain MRI findings had neurological symptoms suggesting brain injury, whereas none of the patients without potential brain injury symptoms had traumatic findings in brain MRI (*P* = 0.003, Table 2). The difference was statistically significant (*P* = 0.028, Table 3) also among the patients with high-energy trauma (motor vehicle accident, pedestrian struck by car, bicycle crash, fall from a height of ≥ 2 m (*n* = 47).

**Table 2:** Occurrence of brain MRI findings in patients with or without potential brain injury symptoms

	Potential brain injury symptoms	No potential brain injury symptoms	*P*-value
Traumatic findings on brain MRI	25	0	0.003^a^
No traumatic findings on brain MRI	112	42	
Non-traumatic findings on brain MRI	18	5	0.83^b^
No non-traumatic findings on brain MRI	119	37	

^a^*X*^2^ = 8.908, df = 1

^b^*X*^2^ = 0.044, df = 1

**Table 3:** Occurrence of traumatic brain findings in patients with a high-energy trauma

	Potential brain injury symptoms	No potential brain injury symptoms	*P*-value
Traumatic findings on brain MRI	14	0	0.028^a^
No traumatic findings on brain MRI	34	13	

^a^Fisher’s exact test

Traumatic findings of the spine and brain MRI were not associated (*P* = 0.289, Table 4); that is, the presence or absence of spinal injuries did not predict brain injuries. Of the 42 patients without potential brain injury symptoms, 14/42 (33%) had traumatic findings on spinal MRI, including fractures and posterior ligamentous complex injuries. All patients with traumatic findings on both spine and brain MRI suffered of cervical spine injury, while none of the eight patients with a thoracolumbar trauma had traumatic findings on brain MRI.

**Table 4:** Traumatic findings on brain MRI and spine MRI

	Traumatic findings on spine MRI	No traumatic findings on spine MRI	*P*-value
Traumatic findings on brain MRI	7	18	0.289^a^
No traumatic findings on brain MRI	29	125	

^a^*X*^2^ = 1.125, df = 1

Non-traumatic findings on brain MRI were reported in 23/179 (13%) patients without association to the potential brain injury symptoms (*P* = 0.83, Table 2). Of these findings, one was a symptomatic infection that was clinically suspected in addition to acute trauma, while 22/179 (12%) were true incidental findings. Of all non-traumatic findings, 2/179 (1%) led to additional treatment. The first patient was clinically suspected of having an acute infection in addition to the injury because the patient had a low-grade fever and mastoid area erythema in addition to headache, nausea, and neck pain after blunt trauma. The MRI revealed acute otitis media complicated with mastoiditis, and the infection was successfully treated. The other patient had a Chiari I malformation with syrinx as an incidental finding. Elective posterior fossa decompression was performed 6 months later. Incidental findings were further evaluated or temporarily followed up in three patients (grey matter heterotopia, large arachnoid cyst, white matter T2-hyperintensities of unknown etiology), but none required specific treatment. None of the non-traumatic brain MRI findings were considered as a predisposing factor to the injury.

## Discussion

The co-occurrence of spinal and brain injury in the pediatric population has been most often studied in the setting of abusive trauma in young children [11–14]. In clinical practice, the brain and spine are often scanned concurrently in accidental trauma, but the diagnostic yield of this practice has not been studied in patients suspected of having a spine injury. In this sample of 179 pediatric patients with a suspected spinal injury, the brain MRI did not yield additional value in patients without symptoms suggesting brain injury (Table 2). This applied also to the patients with high-energy trauma (Table 3); however, the number of patients with high-energy trauma was small. According to the current guidelines, all pediatric patients with high-energy trauma should undergo brain imaging [19]. Patients with high-energy trauma are also more likely to need surgery for various injuries, and thorough exclusion of the brain injury might help in preventing unexpected events during and after general anesthesia.

Even though we were not able to implement the clinical decision rules for pediatric head trauma (PECARN, CATCH, CHALICE) per se, the results are consistent with the proven high utility of these rules in the assessment of these patients [7, 10, 20, 21]. Our findings support the reliance on clinical decision rules regardless of a suspected or confirmed spine trauma.

Given the MRI’s excellent sensitivity in detecting intracranial [22, 23] injuries, our results can also be generalized to more widely used CT. Avoidance of unnecessary CT examinations is particularly important in the pediatric population because ionizing radiation might increase the future cancer risk [24–26], although the magnitude of the risk is still being debated [27]. Avoiding unnecessary radiation is one of the main motivators behind the clinical decision-making rules for pediatric head trauma imaging. None of the three rules (PECARN, CATCH, CHALICE) cover the possible indications of brain MRI but are solely based on head CT as standard imaging. MRI does not expose the patient to ionizing radiation, but as a time-consuming and expensive examination, brain MRI should not be performed without clinical implications, even if the child is already in the scanner because of suspected spinal trauma. Unnecessary scanning may also lead to prolonged anesthesia in younger children. Therefore, even an ionizing radiation-free MRI should not be performed without prompt clinical indications. However, in the future, it would be possible to adjust the guidelines and decision-making rules to favor the use of MRI with head trauma patients without high suspicion of injury requiring emergency surgery. In addition to the benefits of decreased radiation exposure, this would simplify the patient pathway, as the brain MRI is often performed later to search for diffuse axonal injuries and other traumatic findings that are invisible on CT [28].

Incidental findings in pediatric brain MRI are not uncommon and can lead to additional human and economic burden, while incidental findings requiring specific treatment are rare [29]. The overall proportion of incidental findings (13%) and the incidental findings leading to treatment (0.5%) in our study sample align with estimates from previous literature [26].

Our work has several limitations. The most obvious are the inherited biases of a retrospective study. Not all patients with a suspicion of spinal trauma but without symptoms suggesting brain injury underwent brain MRI, and the sample size is small. The clinical variables were not primarily collected in a structured manner; however, it is highly unlikely that any neurological symptoms in pediatric trauma patients have been overlooked by the physician in the emergency department. We could not apply the clinical decision-making rules of pediatric head trauma (PECARN, CATCH, CHALICE) or GCS/pGCS to our study, but our criteria for potential brain injury-related symptoms were lower than the ones in the decision-making rules. Therefore, our detection threshold should be low enough. Overall, these limitations are unlikely to significantly bias our main results.

In conclusion, our results suggest that pediatric trauma patients with suspected or confirmed spinal trauma but without brain injury-related symptoms do not need brain imaging. In the absence of brain injury-related symptoms, it might be possible to refrain from head imaging even in high-impact injuries, but this needs to be confirmed in larger samples. Our findings support the reliance on clinical decision-making rules when assessing the need for head imaging with pediatric trauma patients.

## Data Availability

No datasets were generated or analysed during the current study.
